# Synergistic effect on the visible light activity of Ti^3+^ doped TiO_2_ nanorods/boron doped graphene composite

**DOI:** 10.1038/srep05493

**Published:** 2014-06-30

**Authors:** Mingyang Xing, Xiao Li, Jinlong Zhang

**Affiliations:** 1 Key Lab for Advanced Materials and Institute of Fine Chemicals, East China University of Science and Technology, Shanghai, 200237, China; 2 Department of Chemistry, Tsinghua University, Beijing 100084, P. R. China; 3 These authors contributed equally to this work.

## Abstract

TiO_2_/graphene (TiO_2-x_/GR) composites, which are Ti^3+^ self-doped TiO_2_ nanorods decorated on boron doped graphene sheets, were synthesized via a simple one-step hydrothermal method using low-cost NaBH_4_ as both a reducing agent and a boron dopant on graphene. The resulting TiO_2_ nanorods were about 200 nm in length with exposed (100) and (010) facets. The samples were characterized by X-ray diffraction (XRD), UV-visible diffuse reflectance spectroscopy, X-band electron paramagnetic resonance (EPR), X-ray photoelectron spectra (XPS), transmission electron microscope (TEM), Raman, and Fourier-transform infrared spectroscopy (FTIR). The XRD results suggest that the prepared samples have an anatase crystalline structure. All of the composites tested exhibited improved photocatalytic activities as measured by the degradation of methylene blue and phenol under visible light irradiation. This improvement was attributed to the synergistic effect of Ti^3+^ self-doping on TiO_2_ nanorods and boron doping on graphene.

Since Fujishima and Honda discovered the phenomenon of photocatalytic splitting of water on TiO_2_ electrodes in 1972^1^,^2^,^3^, titanium dioxide (TiO_2_) has emerged as one of the most promising oxide semiconductors and has been employed in diverse applications including air and waste water purifiers, solar energy cells and sensors^4^,^5^. However, the wide band gap and fast recombination of the photoexcited electron-holes of TiO_2_ restrict its use in many practical applications. Therefore, TiO_2_ modification is necessary for improving the optical sensitivity and activity of TiO_2_ in the presence of visible light. Such modifications might include impurity ion doping, noble metal loading, and others^6^,^7^. Among these, impurity doping is an efficient technology for improving the response of TiO_2_ to visible light. However, impurity doping could result in crystal or thermal instability and increased carrier recombination centers^8^.

Owing to its relatively high surface area and special photoelectrochemical properties compared to powder catalysts, many studies on TiO_2_ nanorods have been previously reported. Jun et al.^9^ varied the ratio of a nonselective and a surface selective surfactant (trioctylphosphine oxide and lauric acid, respectively) in dioctyl ether to induce the transformation of TiO_2_ nanoparticles into nanorods dissolved in dioctyl ether. Additionally, Li et al.^10^ synthesized tetragonal faceted-nanorods of single-crystalline anatase TiO_2_ with a large percentage of higher-energy (100) facets. Generally, the previous nanorods were prepared in organic solvent, increasing the tediousness of operational processes and subsequently reducing the working efficiency. In addition, modifications such as doping and controlling the morphology, reduced the amount of TiO_2_ containing Ti^3+^ or an oxygen vacancy and has also been confirmed to exhibit high photocatalytic activity^11^. Our group has previously reported studies on Ti^3+^. For example, Xing et al.^8^,^12^ successfully synthesized Ti^3+^ self-doped TiO_2_ with either NaBH_4_ as the reducing agent or using a vacuum-activated procedure. Both samples exhibited high photo-degradation of organic pollutants. In spite of the research progress achieved on Ti^3+^ and vacancy, there are still some controversies concerning especially the theoretical research on this topic. Rusu et al.^13^ concluded that the photocatalytic activity of rutile increased by vacuum pretreatment through the production of a large amount of anion on the (110) faces. Nevertheless, Hoffmann et al.^4^ ascribed this phenomenon to the reinforced crystallinity achieved via high temperature activation. Meanwhile, Sato et al.^14^ found that heating the material to 500°C induced desorption of surface oxygen and produced many oxygen defects resulting in improved photo-oxidation capacity. On the contrary, Yu et al.^15^ attributed the high photocatalytic activity of TiO_2_ to the existence of Ti^3+^ surface states. This was due to the ability of TiO_2_ to capture photogenerated electrons prior to transferring the electrons to the O_2_ adsorbed on the active sites of surface Ti^3+^, thus reducing the recombination of photogenerated electrons and holes.

On the other hand, after the discovery of an atomic sheet of sp^2^-bonded carbon atoms by Geim et al.^16^,^17^ in 2004, graphene has attracted great interest from both theoretical and experimental scientists. Graphene nanosheets, as two-dimensional (2D) conductors and monolayers of carbon atoms arranged into honeycomb network formations, have attracted attention as a consequence of their unique properties such as elasticity, low density, excellent electrical conductivity, chemical stability and their large surface area^18^,^19^. Additionally, graphene can also potentially act as a support material, allowing semiconductor particles (such as TiO_2_ nanoparticles) to anchor themselves to the surface^20^. Because of this feature, the surface properties of graphene can be widely adjusted by chemical modifications to form composites^7^,^21^. Combining TiO_2_ and graphene into composites is a promising approach to facilitate the effective photodegradation of pollutants under visible light irradiation.

Recently, the fabrication of hybrid materials, such as TiO_2_ loaded onto graphene, has been a popular topic of study. Zhang et al.^17^ synthesized a chemically bonded TiO_2_ (P25)/graphene nanocomposite using a facile, one-step hydrothermal method, affording impressive methylene blue degradation activity. Choi et al.^22^ reported the fabrication of TiO_2_/GR nanocomposites via a facile electrostatic attraction method. Lambert et al.^23^ obtained TiO_2_/GR hybrid materials by mixing graphene oxide (GO) and TiF_4_ followed by ultrasonication and heating before reduction by hydrazine hydrate (HHA) and hydrothermal processing for heightened stability. All of the reported composite hybrids have superior photocatalytic activities compared to other TiO_2_ materials used for the degradation of dyes. Yet, many open problem remain; for example, this process usually gives rise to TiO_2_ aggregation while loading P25 onto GO^24^. While HHA has been widely used in the reduction of GO, it is recognized, however, as an environmental pollutant. Additionally, solvothermal treatment is selective for the epoxy group of GO, leaving the hydroxyl and carboxyl groups unreduced. To mediate these problems, there is strong demand for environmentally friendly reducing agents and novel reduction processes.

Additionally, some doping modifications of graphene in order to improve its electronic properties have attracted a great deal of attention. Tran Van Khai et al.^25^ prepared boron-doped graphene oxides by means of annealing the films at 1100°C. The modified GOs were obtained from suspensions of GO and H_3_BO_3_ in a solution of *N, N*-dimethylformamide (DMF). Similarly, Niu et al.^26^ prepared boron-doped graphene through pyrolysis of graphene oxide with H_3_BO_3_ in an argon atmosphere at 900°C. Each of these experiments adopted high-temperature processes, increasing the economic cost of these methods. Theoretical studies on graphene nanoribbons doped with boron have demonstrated that edge-type as well as substitutional doping can induce half-metallic behavior and that the band gap can be tuned by doping^27^, thus highlighting the potential application of boron-doped graphene (B-GR) in photocatalysis.

Here, we report the preparation of TiO_2_ nanorods in deionized water via a simple one-step hydrothermal method. First, we exposed nanorods with (100) and (010) facets of about 200 nm in length. Next, the composite, consisting of Ti^3+^ self-doped TiO_2_ nanorods were loaded onto the boron-doped graphene sheets. This was successfully achieved using NaBH_4_ as the reducing agent as well as the boron source. The photocatalytic activity of Ti^3+^-TiO_2_/B-graphene composites will also be discussed.

## Results

**Figure 1:**
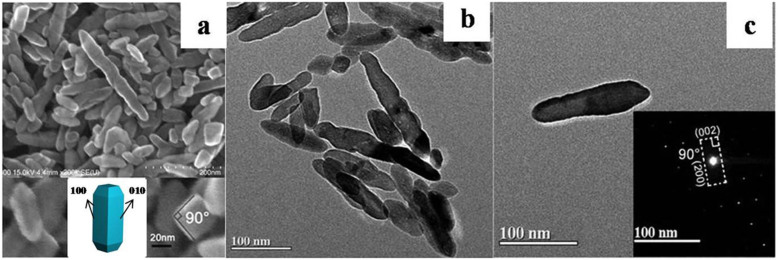
(a) FESEM and (b) TEM images of TiO_2_ nanorods, and (c) the corresponding fast-Fourier transform (FFT). The inset of (a) is the amplified image of TiO_2_ nanorods and a structural moduling image.

**Figure 2:**
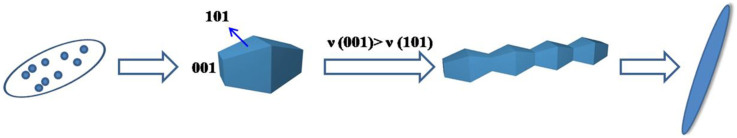
The formation mechanism of TiO_2_ nanorods.

The FESEM and TEM images of TiO_2_ nanorods are presented in Figure 1. The prepared TiO_2_ nanoparticles are shaped like nanorods with lengths in the range of 50–200 nm. It is obvious from the cross-section of the FESEM image (Figure 1a) that the angle between two adjacent sides is 90°. For increased clarification, we set up a structural modeling image. From this image, it is obvious that the nanorod exists with the (100) and (010) facets exposed and at an angle of 90°, which is in agreement with the above result. To further characterize the exposure of the (100) facet, TEM (Figure 1b) and fast-Fourier transform (FFT) (Figure 1c) were performed. The axis direction of the nanorod is parallel to the (002) facet, as determined by FFT, confirming that the nanorod is extended along the (001) direction. Considering the observation of the (200) facet perpendicular to the (002) facet in the FFT image, it can be concluded that the prepared TiO_2_ nanorod exposes the (100) facet. Theoretical studies demonstrated that anatase (100) facets are more active and accordingly exhibit higher catalytic activity than (001) or (101) facets^10^. The mechanism of formation of TiO_2_ nanorods can be explained in the kinetic growth region^9^, shown in Figure 2. The structure of anatase TiO_2_ is tetragonal with the (101) and (001) facets exposed. The added ammonia results in the growth of the (001) facet, resulting in a change in growth velocity, namely, ν (001)> ν (101), ultimately resulting in the formation of nanorods.

**Figure 3:**
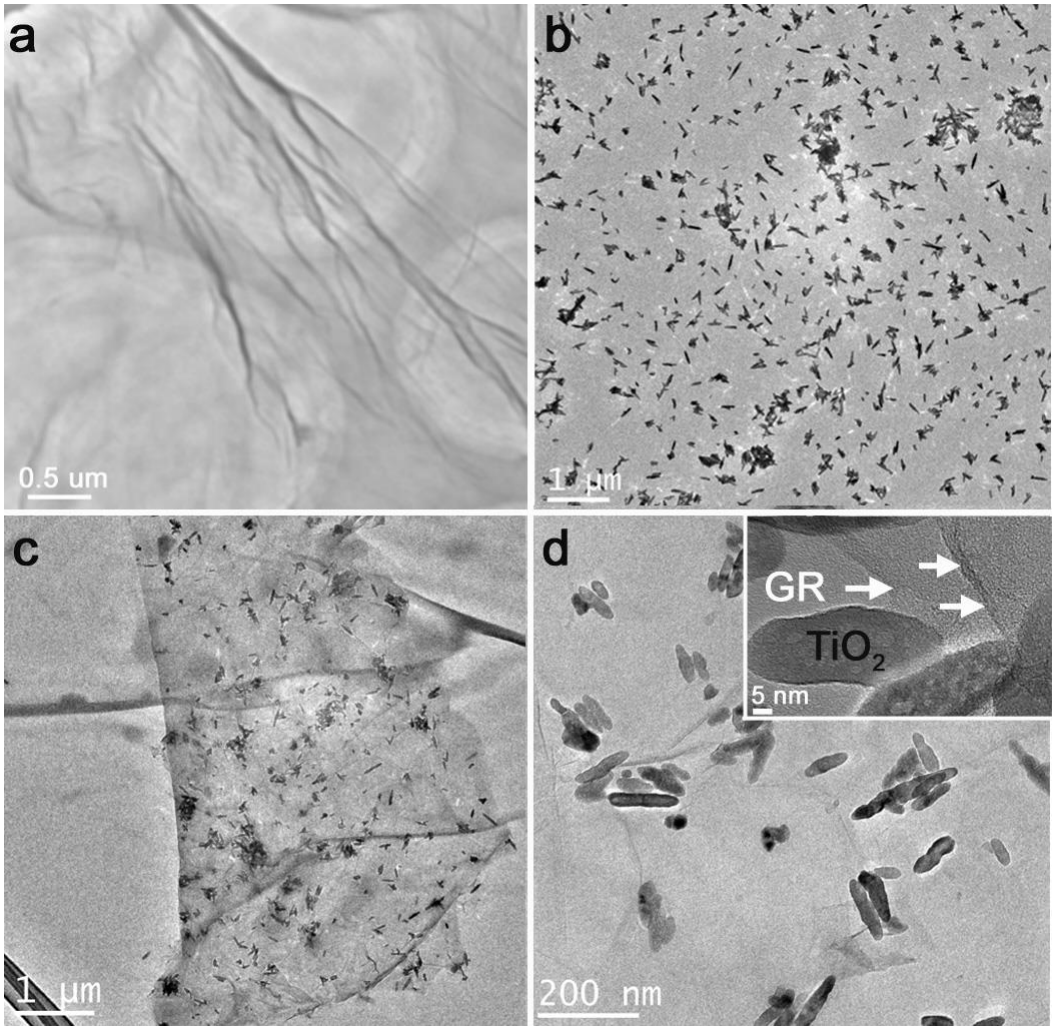
TEM images of (a) graphene oxied, (b) pure TiO_2_ nanorods, and (c, d) 0.1-TiO_2-x_/GR composite.

The loading of TiO_2_ nanorods on graphene sheets was characterized by TEM. Images of pure graphene and TiO_2-x_/GR composites are shown in Figure 3. Figure 3a demonstrates that the prepared sheet-like graphene oxide was a transparent, smooth, and 2D-layered material well suited for the addition of TiO_2_. We intended to load the TiO_2_ nanorods on the wrinkled or edged areas of the GO where carboxyl functional group are likely to be abundant^14^ (Figure 3b–d). Accordingly, the TiO_2_ nanoparticles were covalent bonded to GO, forming a composite favoring the separation of electron-hole pairs (Figure 3d).

To further characterize the composition of the as-prepared samples, we performed Raman spectroscopy (Supplementary Figure S1). The samples exhibited strong peaks at g = 1.978 and g = 1.959, characteristic of Ti^3+^^28^,^29^. The peak corresponding to surface Ti^3+^ is difficult to observe at room temperature due to its instability but it can be inferred that the signal arising from paramagnetic Ti^3+^ centers belongs to bulk Ti^3+^. Additionally, we observed no peaks indicative of surface Ti^3+^ (g = 2.02–2.03) further confirming the interaction between Ti^3+^ and O_2_ to form O_2_^−^30,31. Thus, it can be concluded that sufficient amounts of Ti^3+^ exist in the bulk under conditions using NaBH_4_ as the reducing agent during hydrothermal processing.

**Figure 4:**
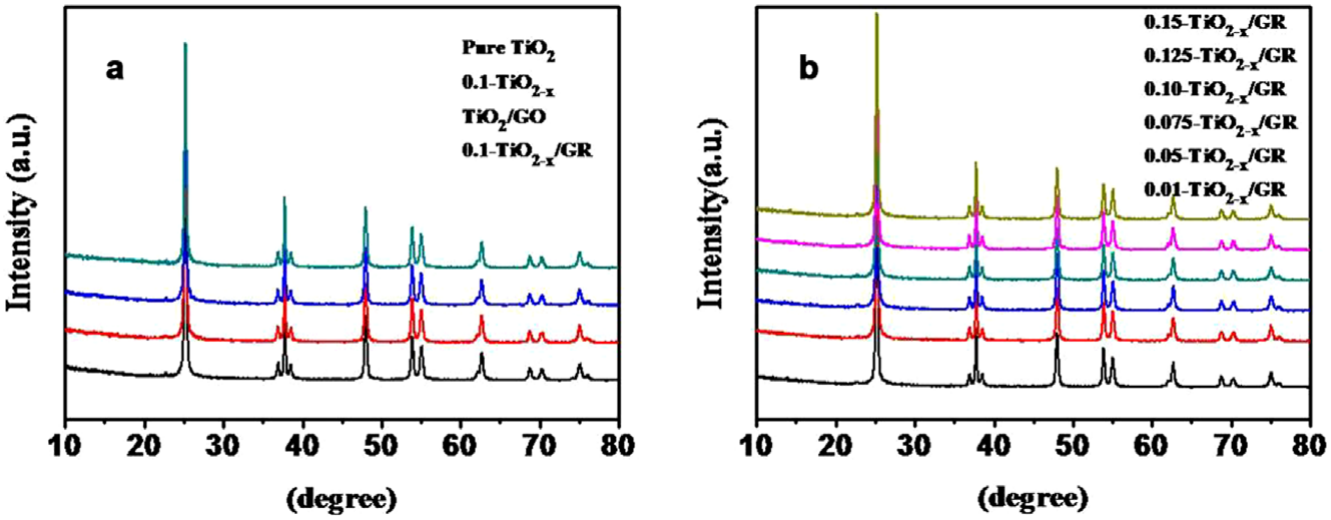
XRD patterns of (a) n-TiO_2-x_/GR samples with adding different amount of NaBH_4_ and (b) 0.1-TiO_2-x_/GR and control blank samples.

XRD patterns of TiO_2-x_/GR composites prepared using different amounts of NaBH_4_ are shown in Figure 4. Well-defined diffraction peaks of the anatase phase structure of TiO_2_ are clearly visible. Diffraction peaks are located at 25.3°, 37.8°, 48.0°, 53.9°, 54.9°, 62.9° and 68.8°, corresponding to the (101), (004), (200), (105), (201), (204) and (116) facets of anatase TiO_2_, respectively (JCPDS No. 21-1272). It can be observed for all composites that increasing amounts of NaBH_4_ do not alter the polymorph of TiO_2_. In all cases, the polymorph can be described as fine anatase crystallites, confirming that the graphene supports are not affecting the phase or structure of TiO_2_. Compared to pure TiO_2_ in Figure 4b, the crystallinity of samples prepared with NaBH_4_ is weakened. This is likely because a large amount of hydrogen gas was evolved during the reaction, resulting in the reduction of Ti^4+^ on the surface to Ti^3+^ and oxygen vacancies during the hydrothermal treatment. These defects inhibited the growth of TiO_2_ nanoparticles, decreasing the crystallinity.

The average crystal size and d-spacing of different samples were determined by XRD using the Scherrer equation as shown in Supplementary Table S1. It can be seen that Ti^3+^ self-doping does not change the phase, however, there is a slight increase in particle size after reduction. It has been reported that boron doping into the lattice tends to lead to lattice distortion^32^, suppressing crystal growth and thereby diminishing the particle size of the catalyst^5^. Therefore, it can be inferred that boron is not introduced into the TiO_2_ lattice here by using NaBH_4_ as the reducing agent. Additionally, “d” space values are similarly unchanged, implying that the doping modification does not change the dimensions of the average unit cell.

**Figure 5:**
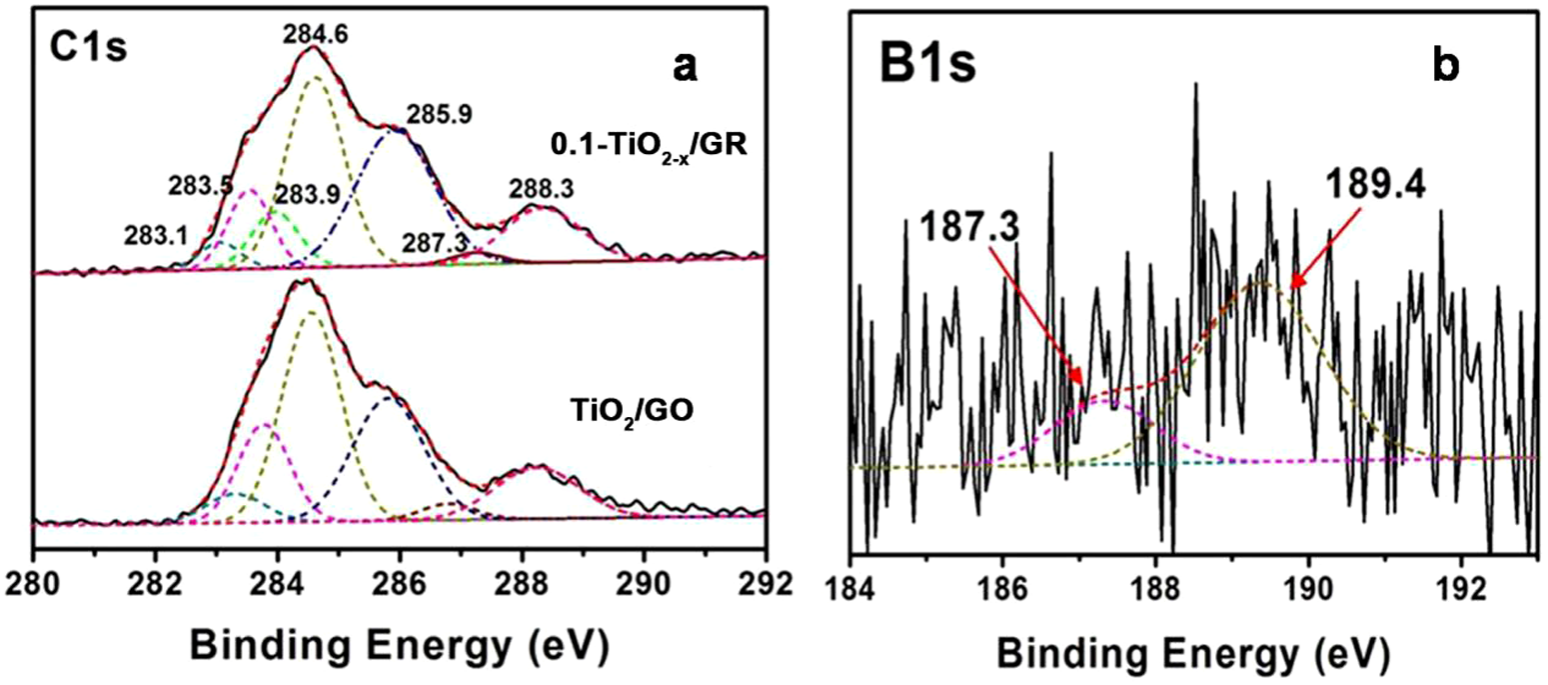
XPS analysis. (a) C1s for 0.1-TiO_2-x_/GR and TiO_2_/GO, and (b) B1s for 0.1-TiO_2-x_/GR composites.

XPS techniques were adopted in order to detect the different chemical states present in TiO_2_/GO and the interaction between GO and TiO_2_. In the C1s core level spectrum (Figure 5), there are six main peaks corresponding to TiO_2-x_/GR composites^7^,^33^,^34^, including: (i) the C of the Ti-O-C corresponding to the interaction of TiO_2_ and graphene (283.1 eV); (ii) sp^2^ C bonds of the graphene skeleton (283.5 eV); (iii) adventitious carbon impurities adsorbed on the surface of sample (284.6 eV); (iv) the C of the C-OH bonds (285.9 eV); (v) the C of the epoxy group (C-O-C, 287.3 eV); and (vi) the C of the carboxyl group (O = C-OH, 288.3 eV). There are large changes in the low field peaks of C1s and the appearance of a new peak at 283.9 eV assigned to the sp^2^ B-C bond^35^. These results indicate that NaBH_4_ was introduced as a reducing agent as well as a boron dopant in the graphene.

The results of the high resolution B1s XPS spectra of the 0.1-TiO_2-x_/GR composite are displayed in Figure 5b, further confirming that the boron has been doped into the lattice of graphene rather than into TiO_2_. The peak at 187.3 eV can be associated with a boron carbide such as C_3_B with boron atoms substituting carbon atoms in the graphene structure^26^. Additionally, there is another new peak at 189.4 eV attributed to C-B bonds resulting from boron supplanting hydroxyl groups on the edges of graphene. It is noteworthy that no peak corresponding to Ti-B bonds appears between 186.0–187.0 eV, demonstrating the absence of boron doping into TiO_2_. The above result is consistent with our previous work^12^.

In order to investigate the presence of Ti^3+^ in TiO_2_ after the addition of NaBH_4_, we performed room-temperature electron paramagnetic resonance (EPR) on NaBH_4_ reduced samples (see Supplementary Figure S2). Strong peaks were observed at g = 1.978 and g = 1.959, characteristic of Ti^3+ 28,29^. The peak of surface Ti^3+^ does not appear at room temperature because of its instability, therefore it can be inferred that the signal of the paramagnetic Ti^3+^ centers belongs to bulk Ti^3+^. In addition, there is no signal peak at g = 2.02–2.03 indicative of surface Ti^3+^, further confirming the interaction between Ti^3+^ and O_2_ to form O_2_^−^30,31. It can be concluded that a large amount of Ti^3+^ exists in the bulk when NaBH_4_ is used as the reducing agent during the hydrothermal process.

Supplementary Figure S3 represents the FTIR spectra of TiO_2_/GO and TiO_2_ before and after addition NaBH_4_. The peak at around 3400 cm^−1^ can be assigned to the vibration of the O-H groups of adsorbed water and Ti-OH groups on the catalyst surface^36^. The intensity of this band is obviously enhanced after the addition of NaBH_4_. The release of hydrogen from NaBH_4_ gives rise to oxygen defects on the TiO_2_ surface during the solvothermal process, helping absorb -OH and H_2_O and thus concentrating hydroxyl groups at the catalyst surface. We also observe peaks corresponding to carbon impurities including saturated and unsaturated C-H and C = O bonds in the range of 2300–3300 cm^−1^. These impurities likely result from solvents present on the sample surface arriving there during the solvothermal process^12^.

The band appearing at about 1600 cm^−1^ of the FTIR spectrum of GO and GR (Figure S3b) can be attributed to the skeletal vibration of the GR sheets^33^, confirming the reduction of GO to GR. By comparison, after reduction, no obvious signals characteristic of oxygen-containing functional groups such as C-O alkoxy, O = C-O carboxyl or -OH hydroxyl can be observed for GR. The peak in the range of 2500–3700 cm^−1^ is sharper and broader for GO compared to GR, likely resulting from residual unreduced -OH and adsorbed water molecules. The curve of GO shows two sharp absorption bands in the range of 1500–2000 cm^−1^ corresponding to the stretching vibration of C = O (1750 cm^−1^) and the bending vibration of O-H (1620 cm^−1^), respectively, but they are not obvious for GR. This indicates that hydrothermal treatment in the presence of NaBH_4_ can effectively result in the reduction of carboxyl and hydroxyl groups and thus the reduction of GO to GR.

The UV-visible diffuse reflectance spectra of pure TiO_2_, 0.1-TiO_2-x_, TiO_2_/GO and TiO_2-x_/GR composites with varying amounts of boron doping demonstrate that the absorption intensity of samples in the visible region modified with Ti^3+^ self-doping is clearly enhanced in comparison to that of pure TiO_2_ (Supplementary Figure S4). This result agrees with our previous work^12^ which also demonstrated that the conversion of Ti^3+^ into TiO_2_ using the vacuum-activated process or NaBH_4_ increased the absorption intensity in the visible region. It should be noted there is an obvious red shift to longer wavelengths in the UV-vis absorption spectra. Considering that band gap narrowing can allow more absorption of visible light and more efficient photogenerated electron transfer, the prepared TiO_2-x_/GR composites are expected to have enhanced photocatalytic performance under visible light irradiation.

## Discussion

**Figure 6:**
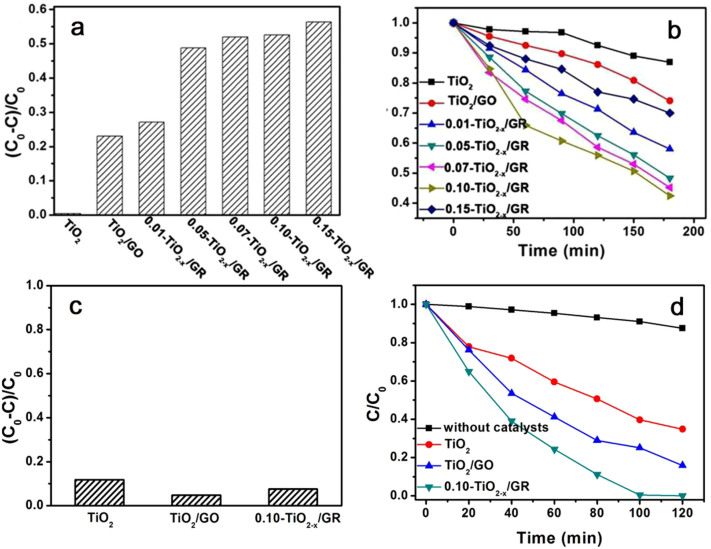
Photocatalytic activities of different catalysts. (a) Adsorption changes in the dark and (b) photocatalytic degradation of MB under visible light of different catalysts. (c) Adsorption changes in the dark and (d) photocatalytic degradation of phenol under the simulated solar light (with an AM 1.5 air mass filter) of different catalysts.

The photocatalytic activity of catalysts in the visible light specturm was investigated for the purpose of demonstrating potential applications. Figure 6a shows the concentration of methylene blue (MB) solution after reaching the adsorption-desorption equilibrium in the dark. Note that the catalyst containing graphene exhibited improved MB adsorption compared to pure TiO_2_. This is likely due to the large π-conjugation system and 2D planar structure of graphene^34^,^36^. Interestingly, with inceased amounts of NaBH_4_, the adsorption capacity of composites was enhanced accordingly. When boron was incorporated into the graphene lattice, the negative surface charge was increased, resulting in a different isoelectric point. This effect is likely to enhance the adsorption of cationic dye molecules. The photocatalytic activities of pure TiO_2_ and composites with different weight ratios of NaBH_4_ were explored by photodegration of 20 mg/L of MB under visible light irradiation (Figure 6b). The photocatalytic activity of TiO_2_ nanorods anchored to B-GR nanosheets was greater than pure TiO_2_. Because of the hydrothermal reduction, TiO_2_ interacted with the graphene surface –OH hydroxyl groups to form Ti-O-C bonds, ultimately resulting incovalently bound TiO_2-x_/GR composites^17^. Several reports found that MB is not appropriate as a model compound for testing visible light induced photocatalytic activity^37^,^38^. In order to fully understand the photocatlytic activity of Ti^3+^-TiO_2_ nanorods/B-graphene composite, the photo-degradation of colorless phenol was measured under simulated solar light irradiation (using an AM 1.5 air mass filter). The results are shown in Figure 6c,d. Unlike the adsorption of MB, the 0.10-TiO_2-x_/GR composite cannot enhance the adsorption of phenol in absense of light (Figure 6c). In addition to the conjugated structure, the surface charges may be another important factor affecting the adsorption of organic molecules on the GR. The phenol's absence of surface charges may explain the poor adsorption onto the surface of TiO_2-x_/GR composites. Recently, many efforts have been made towards the exploitation of TiO_2_-based photocatalysts under intense simulated solar light conditions for industrial purposes. Exmaples include black hydrogen-doped TiO_2_^39^, yellow-vacuumed TiO_2_^40^, and TiO_2_/graphene aerogels^41^. Here, the solar light photocatalytic activities of Ti^3+^-TiO_2_ nanorods/B-graphene composites are investigated to further confirm their photocatalytic performance (Figure 6d). The solar light photocatalytic activity of 0.10-TiO_2-x_/GR for the degradation of phenol is greater than other similar catalysts such as TiO_2_ and TiO_2_/GO. In fact, 0.10-TiO_2-x_/GR can completely decompose of phenol in 100 min under solar light irradiation; a far faster rate than other catalysts. The impurity level between TiO_2_ and GR narrows the band gap and is responsible for the enhanced photocatalytic activity of the Ti^3+^-TiO_2_ nanorods/B-graphene composites. Additionally, the oxygen vacancy and Ti^3+^ produced in the TiO_2_ bulk also decrease the bandgap of TiO_2_.

However, graphene has excellent electron accepting and transporting properties and effectively allows for the transfer of photogenerated electrons from TiO_2_ to the graphene surface. Additionally, since boron atoms have three valence electrons^26^, boron-doped graphene, a kind of p-type semiconductor, could produce abundant photogenerated vacancies for the capture of more electrons and would exhibit clearly improved reduction effectiveness. It can be concluded that the photocatalytic activities of composites depend on the amount of NaBH_4_ added and that the dopant at 0.10 g is optimal. However, when a gross excess of NaBH_4_ was added, the surfaces of TiO_2_ and GR became covered with boron oxide, thus decreasing the number of available active sites on the surface^12^.

**Figure 7:**
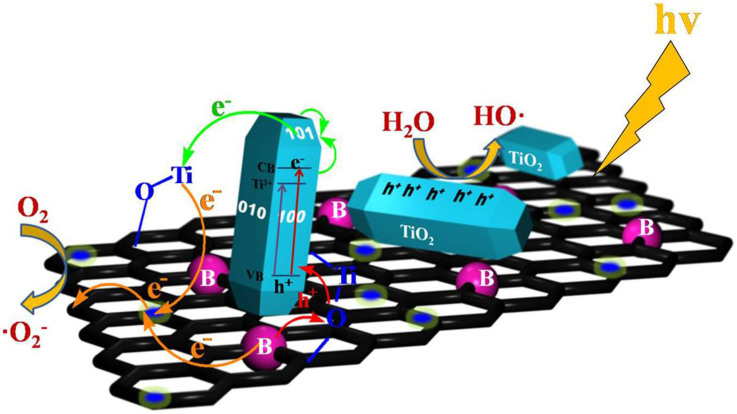
Structural model of energy states. Schematic diagram of the charge transfer of TiO_2-x_/GR composite.

We also explored the mechanism of the photocatalytic activity described here. The impurities introduced by Ti^3+^ self-doping enabled TiO_2_ to respond to visible-light, as shown in Figure 7. As previoulsy mentioned, NaBH_4_ was used as a boron dopant on graphene and the unique p-type semiconductor properties of B-GR enhance hole transfer and effective charge separation. Upon solar-light irradiation, the composite exhibited a significant synergistic effect between Ti^3+^ doping on TiO_2_ and boron doping on graphene. That is, the photogenerated electrons were transfered from the valence band of TiO_2_ to the Ti^3+^ impurity level, narrowing the bandgap of TiO_2_. Also, given that the surface energy of the exposed (100) or (001) facet is relatively high compared to that of the (101) facet, the electrons have the tendency to transfer from (100) or (001) facet to the (101) facet. Finally, the electrons of the nanorod transfered to the graphene surface via the Ti-O-C bonds. Meanwhile, the incremental holes on the B-GR surface were transfered to the valence band, resulting in the effective separation of electron-hole pairs. As a result, electrons were left lying on the graphene sheet and holes on TiO_2_ surface. The electrons can be scavenged by O_2_, in turn producing the superoxide O^2−^ while the positive holes can be trapped by OH^−^ or H_2_O species to produce reactive hydroxyl radicals^42^. All of the reactive radicals are induced by the synergistic effects of Ti^3+^ doping on TiO_2_ and boron doping on graphene, resulting in powerful oxidizing agents for the degradation of dyes and phenols.

In summary, TiO_2-x_/GR composite photocatalysts consisting of Ti^3+^ self-doped TiO_2_ nanorods decorated on boron doped graphene sheets were successfully synthesized via a simple, one-step, hydrothermal method in which low-cost NaBH_4_ was introduced as a reducing agent while simultaneously affording boron as a dopant. The prepared TiO_2_ nanorods were about 200 nm in length with exposed (100) and (010) facets. The prepared catalysts were anatase crystallites with high photocatalytic activity under visible or solar light irradiation. The samples containing 0.10 g NaBH_4_ exhibited better MB adsorption and displayed the overall greatest efficiency in the degradation of MB and phenol. The high solar light-dependant activity was attributed to the synergistic effect between Ti^3+^ self-doped TiO_2_ and boron-doped graphene.

## Methods

Preparation of graphene oxide (GO). GO was synthesized from natural graphite flakes by a modified Hummers method^43^,^44^. The synthesis method is as follows: Flake graphite (1 g) and NaNO_3_ (0.5 g) were added into cold (0°C) concentrated H_2_SO_4_ (23 mL) in a flask. 3 g of KMnO_4_ was slowly added to the flask under vigorous stirring and the temperature was kept below 20°C. The mixture was stirred at 35°C for 30 min and then diluted with de-ionized water (40 mL), causing a gradual increase in temperature to 98°C. The suspension was kept at 98°C for 15 min. Subsequently, 140 mL of deionized water and 10 mL of 30 wt% H_2_O_2_ solution were slowly added into the mixture, after which the suspension turned bright yellow and evolved bubbles. The mixture was filtered and washed several times with 5% HCl solution to remove residual salt and impurities^45^,^46^,^47^. The resulting solid was dried *in vacuo* at 60°C overnight and finally ground into powdered GO.

Preparation of TiO_2_ nanorods. 40 mL of deionized water was added to 2.0 g of titanium sulfate in a cylindrical vessel and stirred for 30 min before the slow addition of 20 mL of ammonia under vigorous stirring. Stirring was continued for 1 h. Then the cylindrical vessel was sealed in a Teflon-lined autoclave and hydrothermally treated at 180°C for 24 h. As the autoclave cooled to room temperature under ambient conditions, the resulting suspension was centrifuged before being washed with deionized water for five times. TiO_2_ nanorods were obtained by drying at 60°C in a vacuum oven.

In order to remove impurities, the TiO_2_ powder was calcinated at 500°C for 60 min with a heating rate of 2°C/min and the final sample was denoted as Pure TiO_2_.

Preparation of Ti^3+^ doped TiO_2_ nanorods/boron doped graphene composite photocatalyst. 0.03 g of GO was mixed with 70 mL of deionized water before ultrasonic dispersion for 1 h. Before adding specific and different amounts of NaBH_4_, 0.5 g of pure TiO_2_ was added and the suspension was stirred for 2 h. Subsequently, the mixture was hydrothermally treated at 150°C for 12 h. After it cooled to the room temperature, the precipitate was collected by centrifugation for 40 min before the addition of 50 mL hydrochloric acid (1 M), followed by stirring for an additional for 3 h. The HCl solution was used to remove the by-products of boron oxides^12^. The resulting solution was washed with deionized water five times and the solid was dried *in vacuo* at 60°C for 12 h. The final sample was denoted as n-TiO_2-x_/GR, where n is the weight of NaBH_4_, chosen as 0.01 g, 0.05 g, 0.075 g, 0.1 g, 0.125 g and 0.15 g.

For comparison, control samples were prepared in the absence of NaBH_4_ or GO according to the above procedure. These were denoted as TiO_2_/GO and 0.1-TiO_2-x_ (“0.1” denoted the weight of NaBH_4_), respectively.

Characterization. X-ray diffraction (XRD) measurements were performed with a Rigaku Ultima IV (Cu Kα radiation, λ = 1.5406Å) in the range of 10–80° (2θ). The morphologies were characterized by transmission electron microscopy (TEM, JEM2000EX) and scanning electron microscopy (SEM, JEOL JSM-6360 LV). The instrument employed for X-ray photoelectron spectroscopy (XPS) studies was a Perkin-Elmer PHI 5000C ESCA system with Al Kα radiation. The shift of the binding energy was referenced to the C1s level at 284.6 eV as an internal standard. The X-band EPR spectra were recorded at room temperature (Varian E-112). The Fourier transform infrared (FTIR) spectra were recorded with KBr disks containing the powder sample with an FTIR spectrometer (Nicolet Magna 550). Raman spectra measurements were recorded with an inVia Reflex Raman spectrometer with 524.5 nm laser excitation. UV-vis diffuse reflectance spectra (DRS) were obtained with a SHIMADZU UV-2450 spectroscope equipped with an integrating sphere assembly and using BaSO_4_ as reflectance sample.

Photocatalytic Measurements. The visible light photocatalytic activity was measured by analyzing the degradation of methyl blue (MB) (20 mg/L). Solar light photocatalytic activity was measured by analyzing the degradation of phenol (10 mg/L). 0.06 g of prepared sample was added into a 100 mL quartz photoreactor containing 60 mL of MB/phenol solution. After ultrasonication for 1 min, the suspension was stirred in the dark for an hour to achieve adsorption-desorption equilibrium on the catalyst surface. A 500 W tungsten halogen lamp equipped with a UV cutoff filter (λ>420 nm) was used as a visible light source and the distance between the light and the reaction tube was fixed at 10 cm. The lamp was cooled with flowing water in a quartz cylindrical jacket around the lamp, and the ambient temperature was maintained during the photocatalytic reaction. A 300 W Xe lamp with an AM 1.5 air mass filter was used as a simulated solar light source. The mixture was stirred for 60 min in the dark in order to reach the adsorption–desorption equilibrium. At regular irradiation intervals, the dispersion was sampled (ca.5 mL), centrifuged, and subsequently filtered to remove the photocatalyst. The resulting solution was analyzed by checking the maximum absorbance of the residual MB/phenol solution with a UV-vis spectrophotometer (Varian Cary 100) at 660/270 nm.

## Author Contributions

M.X. and J.Z. conceived and designed the experiments. M.X. and X.L. prepared the samples and performed characterization. M.X., X.L. and J.Z. were mainly responsible for preparing the manuscript. All the authors discussed the results and reviewed the manuscript.

## Supplementary Material

Supplementary Information Supplementary information
