# Supplementary material for: Synergistic effect on the visible light activity of Ti3+ doped TiO2 nanorods/boron doped graphene composite
Source: Sci Rep. 2014 Jun 30;4:5493. doi: 10.1038/srep05493 (PMC4074785; doi:10.1038/srep05493)
Supplement: Supplementary Information [file srep05493-s1.doc]

**Synergistic effect on the visible light activity of Ti^3+^ doped TiO_2_ nanorods/boron doped graphene composite**

Mingyang Xing^1^, Xiao Li^1^, and Jinlong Zhang^1,2^*

1 Key Lab for Advanced Materials and Institute of Fine Chemicals, East China University of Science and Technology, Shanghai, 200237, China

2 Department of Chemistry, Tsinghua University, Beijing 100084, P. R. China

* E-mail: jlzhang@ecust.edu.cn

Author contributions

Mingyang Xing & Xiao Li contributed equally to this work


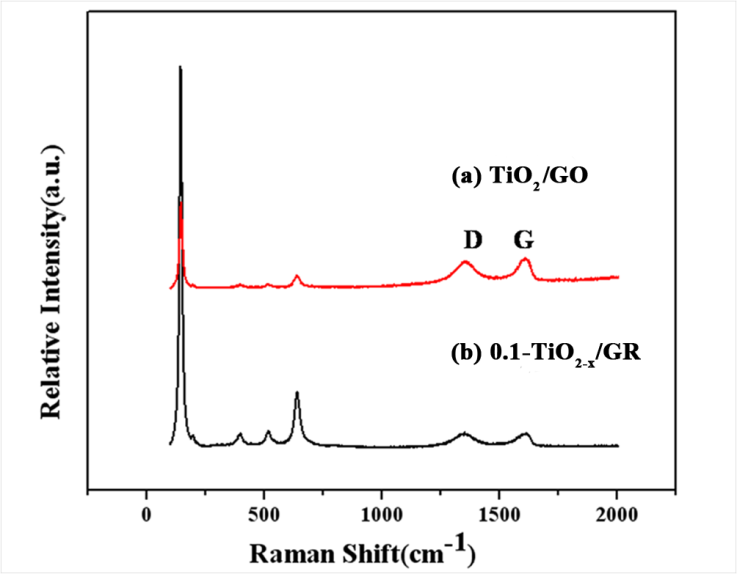


**Fig. S1** Raman spectra of (a) TiO_2_/GO (without reduction) and (b) 0.1-TiO_2-x_/GR.


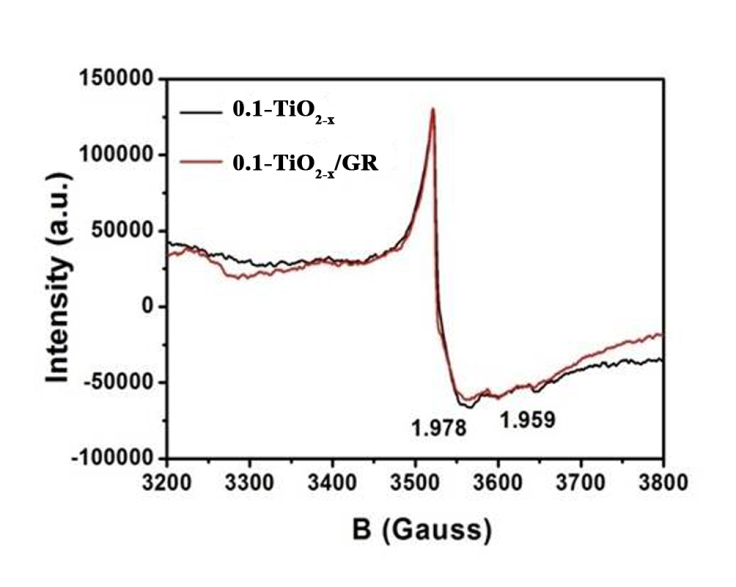


**Fig. S2** EPR spectra of 0.1-TiO_2-x_/GR and 0.1-TiO_2-x_.


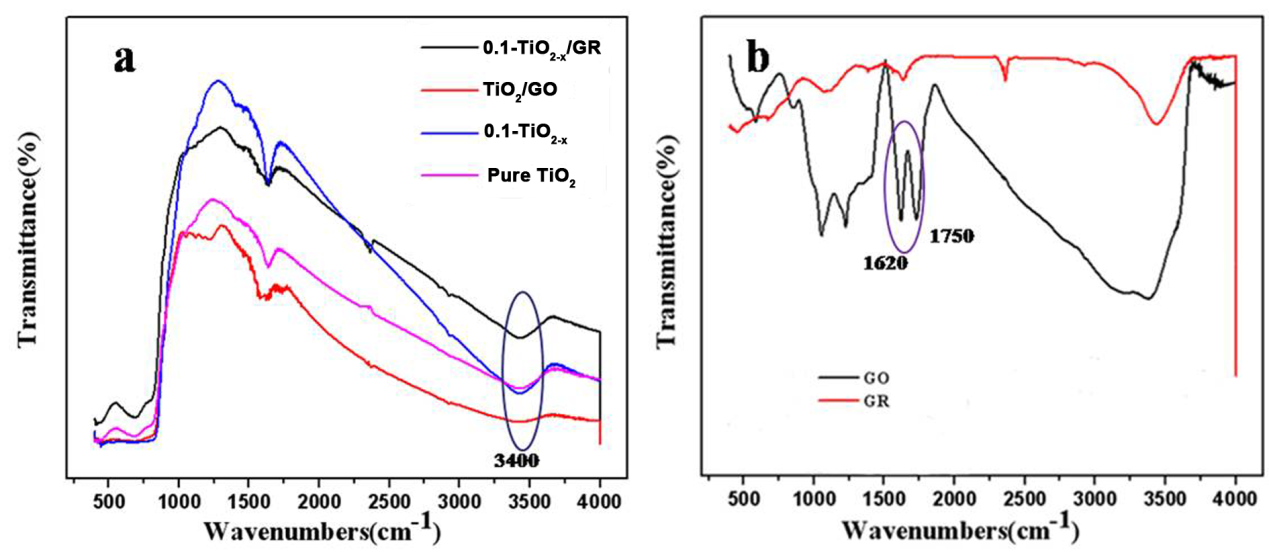


**Fig. S3** FTIR spectra of (a) TiO_2_/GO and TiO_2_ before and after adding NaBH_4_ and (b) GO and GR.

**
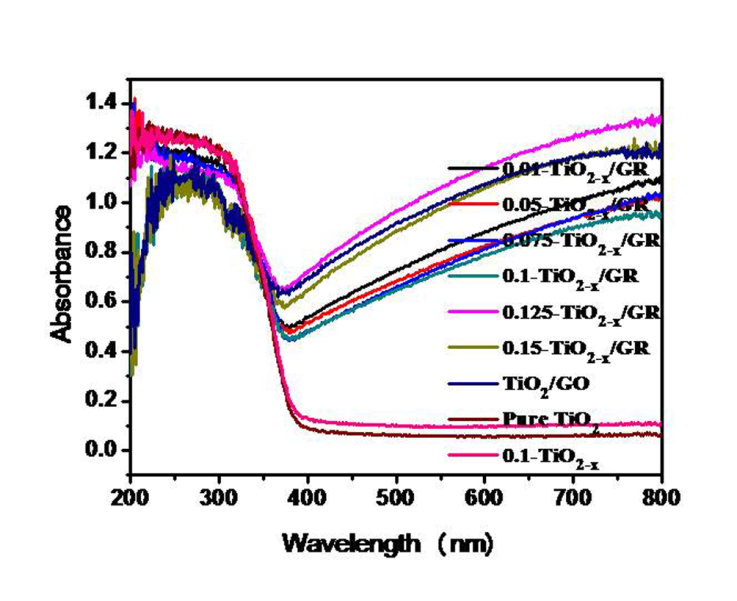
**

**Fig. S4** The UV-vis diffuse reflectance spectra of pure TiO_2_, 0.1-TiO_2-x_, TiO_2_/GO and 0.15-TiO_2-x_/GR, 0.1-TiO_2-x_/GR, 0.07-TiO_2-x_/GR, 0.05-TiO_2-x_/GR, 0.01-TiO_2-x_/GR composites.

**Table S1 Structure parameters of samples.**

| Samples | Particle Size (nm) | d-spacing (Å) |
| --- | --- | --- |
| 0.01-TiO_2-x_/GR | 36.6 | 3.5 |
| 0.05-TiO_2-x_/GR | 37.8 | 3.5 |
| 0.075-TiO_2-x_/GR | 37.5 | 3.5 |
| 0.1-TiO_2-x_/GR | 37.8 | 3.5 |
| 0.125-TiO_2-x_/GR | 38.7 | 3.5 |
| 0.15-TiO_2-x_/GR | 36.9 | 3.5 |
| TiO_2_/GO | 35.3 | 3.5 |
| 0.1-TiO_2-x_ | 35.2 | 3.5 |
| Pure TiO_2_ | 36.8 | 3.5 |
